# Skeletal injuries after cesarean section — a rare differential diagnosis of child abuse

**DOI:** 10.1007/s00414-023-02965-8

**Published:** 2023-02-13

**Authors:** L. Küppers, C. Schaffer, M. Helbig, S. Ritz-Timme

**Affiliations:** 1 Institute of Legal Medicine, University Clinic Düsseldorf, Düsseldorf, Deutschland; 2 Clinic for Gynecology and Obstetrics, University Clinic Düsseldorf, Düsseldorf, Deutschland

**Keywords:** Birth injury, Obstetric fracture, Cesarean section, Long bone fracture, Skull fracture

## Abstract

Birth-related fractures are an important differential diagnosis of child abuse in early infancy. While fractures associated to vaginal deliveries are well known, cesarean section is not necessarily known to cause such injuries. Nevertheless neonatal fractures have been described after cesarean sections. To give an overview over the frequency and typical locations of such fractures, the appearance of symptoms and the timespan until diagnosis, a literature research was conducted via Google scholar and Pubmed, using the key words “cesarean section” and “fractures”. Birth-related fractures after cesarean sections are rare but can occur, with the long bones being particularly affected. Therefore, birth injuries should always be considered in the forensic medical assessment of fractures in early infancy, even after cesarean section. To enable a differentiation between birth trauma and physical abuse, birth and operation records should be checked for surgical manoeuvres, possible difficulties during the procedure or other risk factors. Birth-related fractures are usually detected early; in rare cases, the diagnosis is made only weeks after birth.

## Introduction

Birth injuries are one of the differential diagnoses that regularly have to be considered when evaluating fractures suspicious of child abuse in an infant. A reliable forensic evaluation of the relevant findings is of considerable importance, both for the assessment of a possible endangerment of the child’s welfare and with regard to legal consequences. In the case of abuse-related fractures in a child without bone disease, it must generally be assumed that the trauma was very severe whereas birth related injuries can result from — not always avoidable—mechanical forces during birth. Accidental trauma as a cause of fractures in infancy is rare because of the restricted mobility of infants and the elasticity of the child’s bones. Usually an accidental trauma only comes into question as a differential diagnosis if a correspondingly significant and otherwise suitable event is described [1].

The general incidence of birth-related fractures — independent of the delivery mode — was given in a recent population-based study with 2.9 per 1000 live births [2]. The most common birth-associated fractures are clavicle fractures, which probably result from compression of the anterior part of the fetus’ shoulder against the maternal symphysis [3, 4] or can occur due to management of shoulder dystocia [5]. Skull fractures and long bone fractures are much less common but also possible, while rib fractures are considered extremely rare birth injuries [2, 6]. However, a possible higher number of unreported cases of rib fractures should be considered, since they are mostly clinically silent and can be overlooked in X-ray images if a callus has not (yet) formed [2].

While the causation of fractures in vaginal deliveries is easy to understand against the background of the child’s passage through the mother’s bony pelvis, this does not necessarily apply to fractures in cesarean sections (c-sections). Nevertheless, neonatal fractures have also been described in the context of c-sections [7, 8]. In forensic medical casework, a differentiation between a c-section–related fracture and fracture due to child abuse can therefore become relevant.

In the present article, essential findings on neonatal fractures after c-sections are summarized in the form of a narrative review of the relevant literature.

## Methods

A selective literature search was carried out via Pubmed and Google Scholar for the keywords “cesarean section” and “fractures” taking into account publications of the last 40 years. The publications identified were only included in the further evaluation if they contained information on birth-related fractures in newborns specifically after c-section. Studies or case reports involving fractures in newborns with bone disease such as osteogenesis imperfecta and other than English or German language publications were not included. When evaluating the remaining work, the following points were addressed in particular: frequency of fractures, types of fractures, underlying mechanisms, length of time until the occurrence of clinical abnormalities and/or until the radiological diagnosis.

## Results and discussion

The literature research resulted in a total of 35 evaluable and thematically suitable papers on bony injuries after c-section, including 11 clinical and 2 population-based studies as well as 22 case reports. Table 1 provides an overview of the publications included in this review. In the following, the essential contents of the relevant publications are summarized.

**Table 1:** Birth traumatic fractures after c-section: fracture types and time spans in the relevant publications

Publication	Type of publication	Number of cases with fractures after c-section	Diagnosed fracture locations	Time span after birth until the appearance of first symptoms	Time span after birth until the radiological diagnosis of the fracture
Alexander et al. (2006) [9]	Prospective cohort study	25	Skull, clavicle, long bones	No information given	No information given
Al-Habdan (2003) [10]	Retrospective study	8	Clavicle, humerus, femur, radial bone	No information given	Immediately
Bombah et al. (2021) [11]	Case report	1	Femur	Immeditately	Immediately
Capobianco et al. (2013) [12]	Case report	1	Femur	1 day	1 day
Cebesoy et al. (2009) [13]	Case report	1	Femur	2 days	2 days
Choi et al. (2016) [14]	Retrospective study	19	Clavicle	No information given	No information given
Dave et al. 2020 [15]	Case report	1	Femur	Immediately	Immediately
Erdem et al. (2016) [16]	Case report	1	Femur (bilateral, newborn with Myelomeningocele)	Immediately	Immediately
Farikou et al. (2014) [17]	Case report	1	Femur	Immediately	Immediately
Garcia Garcia et al. (2002) [18]	Case reports	3	Femur	Immediately	Immediately
Hariharan et al. (2020) [19]	Retrospective study	6	Transphyseal humeral separation	No information given	No information given
Högberg et al. (2020) [2]	Population based study	122	Skull, clavicle, femur, others	No information given	Immediately, at the latest ‘beyond the neonatal period’
Jain und Bielski (2001) [20]	Case report	1	Femur	2 days	3 days
Kamaci et al. (2014) [21]	Case report	1	Transphyseal humeral separation	1 day	1 day
Kanai et al. (2018) [22]	Case report	1	Femur	8 days	9 days
Kim et al. 2015 [23]	Case report	1	Femur	Incidental finding	Immediately
Lysack und Soboleski (2003) [24]	Case report	1	Classic metaphyseal lesion of the femur and tibia	Immediately	Immediately
Matsubara et al. (2008) [25]	Case report	1	Femur	1 day	1 day
Mileto et al. (2014) [26]	Case report	1	Tibia	Immediately	Immediately
Moczygemba et al. (2010) [27]	Population based study	Exact number of fractures after c-section not given	Diverse	No information given	No information given
Morris et al. (2002) [28]	Prospective study	5	Femur	No information given	1 day, at the latest 21 days
Nadas und Reinberg (1991) [29]	Retrospective study	10	Skull, clavicle, long bones	Immediately, at the latest 14 days	Immediately, at the latest 14 days
O’Connell und Donoghue (2007) [30]	Retrospective study	3	Classic metaphyseal lesions of the femur	2 days	2 days
Papi et al. (2019) [31]	Case report	1	Femur	1 day	1 day
Patonay et al. (2010) [32]	Case report	1	Skull	No clinical appearance	Diagnosed at autopsy at an age of 4.5 months
Pavone et al. (2020) [33]	Retrospective study	5	Femur	No information given	At the latest 4 days
Rahul et al. (2017) [34]	Case report	1	Humerus (bilateral), Femur	2 days	2 days
Rasenack et al. (2010) [7]	Case reports	4	Skull, humerus, femur	Immediately, at the latest 2 days	Immediately, at the latest 2 days
Rieger-Fackeldey et al. (1999) [35]	Case report	1	Skull drepression	Immediately	Immediately
Sherr-Lurie et al. (2010) [36]	Retrospective study	5	Humerus	Immediately	Immediately
Singla et al. (2021) [37]	Case report	1	Femur	1 day	1 day
Skajaa et al. (1987) [38]	Case report	1	Skull	Immediately	Immediately
Toker et al. [39]	Retrospective study	12	Femur	4 days	10 days
Ulici et al. (2022) [8]	Retrospective study	36	Clavicle	No information given	No information given
Vasa and Kim (1990) [40]	Case report	1	Femur	Immediately	Immediately

### Frequency of fractures in c-sections

Bony injuries after c-section are rare in children without bone disease, but are repeatedly described in the literature [7].

In a 2006, prospective cohort study that included 37.110 cesarean deliveries at thirteen US university hospitals, injuries in newborns were identified in 418 cases (1.1%) [9]. Only 25 cases (0.07%) involved fractures, including eleven clavicle fractures (0.03%), eight long bone fractures (0.02%), and six skull fractures (0.02%). According to the authors of this study, the highest risk for birth fractures as well as for birth injury in general occurs when c-sections have to be performed under time pressure, such as after unsuccessful attempts of a forceps or vacuum delivery. In contrast, elective repeat c-sections are associated with a lower risk [9].

Nadas and Rheinberg [29] reported 28 cases of birth-related bone fractures. Ten of these fractures (35.7%) were diagnosed after c-sections. Delivery by c-section, breech presentation and preterm birth were identified as specific risk factors for fractures of the long bones. Remarkably, all femoral fractures (*n* = 6, 21.4%) in this study were diagnosed after c-section. In contrast, only one in seven clavicle fractures occurred during c-section.

Morris et al. [28] found an incidence of 0.13 femoral fractures per 1.000 in their prospective study of 55.296 live births. In this study, seven children had femur fractures (0.01%). Five of the affected newborns (0.01%) had been delivered by c-section, four of them (0.01%) out of breech position. The fractures in these newborns were all spiral fractures.

Toker et al. (2009) [39] undertook a retrospective study taking into account 221.939 deliveries including 38.990 c-sections. They reported an incidence of 0.31 femoral fractures per 1.000 newborns delivered by c-section. In their study, it was found that a c-section increased the risk of femoral fractures compared to vaginal births (odds ratio 11.26, confidence interval 3.97–31.97).

Pavone et al. [33] performed an observational study including 15.628 children hospitalized at the age of under one month for whatever reason. They found eight (0.05%) newborns hospitalized for femoral fracture with five (0.03%) of them having been born by c-section. They authors stated that c-sections could be a risk factor for femoral fractures.

Sherr-Lurie et al. [36] studied the incidence and characteristics of humeral fractures in neonates shortly after birth. Nine humeral fractures (0.01%) were identified among 92.882 live births (incidence: 0.09 per 1.000 live births). A c-section had been performed in five of these cases (0.01%), with a proportion of 20% c-sections among all births.

Moczygemba et al. [27] found in a population-based US study including 8.176.523 singleton live births that the overall risk of injury in c-sections is lower (*n* = 412.066, 17.1%) than in vaginal births (*n* = 1.701.679, 29.5%). However, there was an equal proportion of skeletal injuries in their study (c-section: *n* = 7.966, 0.33%; vaginal delivery: *n* = 19.592, 0.34%), with clavicle fractures being listed separately.

Choi et al. [14] found an incidence of 0.05% clavicle fractures (*n* = 18) in their study taking into account 36.286 c-sections. Ulici et al. [8] studied 136 newborns with clavicle fractures, 36 (26.5%) of whom had been delivered by c-section (with 25 elective c-sections, 18.4%). In any case, clavicle fractures are much more common after vaginal births than after c-sections [27].

### Typical locations of c-section–related fractures and underlying mechanisms

Table 1 gives an overview over the reported typical locations of c-section–related fractures.

There is a number of case reports on c-section–associated fractures of the femoral shaft, predominantly as a result of c-sections in breech pregnancies [7, 12, 13, 15–17, 20, 22, 25, 31, 37, 40]. Rahul et al. [34] presented a case with bilateral humerus and unilateral femur fracture in a child without bone disease. Inappropriate surgical manoeuvres such as energetic pulling, insufficient uterine incisions, and poor uterine relaxation were assumed to be the causes of such injuries [34]. However, preceding attempts of external cephalic version, twin pregnancies, fetal micro- and macrosomia, prematurity [11, 18, 23, 41] as well as breech presentation [29] have also been identified as risk factors. In the case of spiral fractures, applied torsional forces due to difficulties during the development from the uterus can be considered as the underlying mechanism [28].

Mileto et al. [26] reported a fracture of the tibia after an emergency c-section performed due to failure of birth progress and four unsuccessful attempts of vacuum delivery. Lysack und Soboleski [24] presented a case of classic metaphyseal lesions (CML) of the distal femur and proximal tibia after a c-section with previous attempts of an external cephalic version. The authors attributed such fractures to torsional or shearing forces during the development from the uterus [24]. O’Connell und Donoghue [30] described three cases of neonates with CML of the distal femur after uncomplicated cesarean deliveries (without prior attempts of an external cephalic version). These case reports are of great importance for forensic medical assessment, since CML are generally considered to be highly specific for child abuse [42].

Humeral fractures after c-section have been described as a result of energetic traction during delivery from the uterus [34]. Sherr-Lurie et al. [36] pointed out that fractures of the distal humeral epiphysis can sometimes be overlooked radiologically as long as there is no callus formation. Furthermore, case reports on transphyseal separations of the humerus after cesarean section have been published [19, 21]. This injury is presumed to be a result of excessive traction to the forearm during c-section [21].

Skull fractures after c-section are very rare and predominantly occur when forceps or vacuum delivery attempts preceded the c-section [9, 29]. In this respect, they not necessarily represent results of the surgical manoeuvres [9]. Nevertheless, some cases have been described in which compression of the head during development from the uterus resulted in skull fractures: Skajaa et al. [38] published the case of an impression fracture of the skull as a result of digital pressure during a difficult development of the fetal head. Rasenack et al. [7] observed a skull fracture after an urgent c-section with forced pushing of the child’s head out of the pelvis.

Fractures of the clavicle sometimes even occur after elective c-sections [9]. The underlying mechanisms are still unclear, but a high birth weight and an advanced maternal age were found to be risk factors [14].

In forensic medical casework, medical records such as birth or operation reports should be analysed with special regard to risk factors, delivery techniques suitable as a cause for trauma or the description of a difficult development of the child from the uterus.

### Real time witnessing, first appearance of symptoms, and timespan until diagnosis

There are rare cases published in which the origin of the fracture was witnessed in real time by noticing a “cracking” sound or the feeling of a “snap” during delivery of the child from the uterus [25, 40]. In one case of a perinatal skull fracture the authors described that the surgeon had felt a “ping pong ball crack” under his index finger during delivery of the head although he did not apply much force to it [38]. A real time diagnosis of obstetric fractures is yet a rarity.

Nevertheless in the vast majority of cases, birth-related fractures are diagnosed immediately or at the latest within the first two days after birth [12, 16]. An overview of the stated time spans in relevant publications is given in Table 1.

Rather unspecific clinical signs of a fracture are irritability, feeding problems and increased crying, whereas reduced mobility of the affected body part as well as palpable step formation or crepitation represent more specific indications of a bone injury [12, 29].

A special group of cases that have been reported were newborns who presented with (non-specific) clinical symptoms from birth, but who nevertheless had a delayed diagnosis of fractures, for example [39]. The presence of such bridging symptoms should be checked in the forensic medical assessment as they can be an indication of birth-related fractures.

As is shown in Table 1, some cases have been described in the literature in which birth associated fractures were diagnosed only at the age of a few weeks. In fact, such fractures can be clinically silent, especially in the case of linear skull fractures and fractures without loose bone fragments or step formation [2]. According to Morris et al. one case in their study was not diagnosed with a presumed birth-related femur fracture until 21 days after birth [28]. Högberg et al. [2] found that while 94.1% of all birth-related fractures in their study were diagnosed in the early neonatal period, 1.1% were only diagnosed beyond the neonatal period. Patonay et al. [32] described the case of a 4.5 month old twin boy who died unexpectedly. During autopsy a healing linear fracture of the skull as well as a purple-red, circular discoloration over the calvarium were observed and child abuse was suspected. Since neither the autopsy nor the following histological examination showed any signs of recent trauma but lesions in a later healing stage, the authors assumed the injuries to be most likely birth related.

In forensic medical casework, it should be emphasized that obstetric fractures diagnosed only weeks after birth are rare. In addition, from a forensic medical perspective, the question arises as to how a later occurrence of the bone injuries, possibly as a result of a subsequent child abuse, could even be ruled out in the cases mentioned above. Högberg et al. [2] described how in some of the cases the diagnosis of child abuse was made in the course of their study, but it does not go into whether abuse could be ruled out in the other cases. Morris et al. [28] did not explain this in detail either. These points are a frequent problem in forensic medical science. On one hand, large cohort studies and big clinical databases are urgently needed to reach a certain level of scientific evidence; on the other hand, the evaluation of the single case is hardly controllable in large studies. Forensic medical experts need to keep in mind that the results of such studies can only be used carefully. At last, the question up to which age a birth related fracture has to be considered cannot finally be answered. To our knowledge, the case report of Patonay et al. (2010) is the case with the longest interval between birth and an assumed obstetric fracture (4.5 month) published, but it remains unclear if this fracture really was birth related.

### Differential diagnoses

When assessing fractures in newborns, obstetric injuries are not the only possible differential diagnosis to child abuse. In any case of a suspected child abuse with a bone injury in an infant an underlying bone disease should be ruled out. Medical guidelines such as the German AWMF S3( +) guideline on child abuse and neglect [43] give valuable information on what kind of diseases are to consider and how to exclude them. This does not necessarily mean that every birth-related fracture must be followed by a diagnostic screening for bone diseases.

It has to be mentioned that bone injuries diagnosed in newborns not always occur at birth. They can also represent prenatal alterations as for example a so called “ping-pong” deformity of the fetal skull which can be a result of in utero pressure on the head [44]. The possible reasons for such a deformity are manifold and are ranging from deformities of the maternal bony pelvis to the fetuses own extremities being stuck between the maternal pelvis and the fetal skull. Furthermore maternal accidents during pregnancy can be causal [35].

## Conclusion

Birth-related fractures after c-sections are rare but can occur, with the long bones being particularly affected (especially the femur). Therefore, birth injuries should always be considered in the forensic medical assessment of fractures in early infancy, even after c-section. This requires a careful inspection of the birth and medical records, with particular attention being paid to information about difficulties in the development of the fetus from the uterus. Regarding the differentiation between birth trauma and physical abuse, it has to be considered that cases with an (initially) clinically silent course have been described — even if it is not always clear from the clinical literature how reliably abuse has been ruled out. Nevertheless, such clinically silent cases are very rare; birth-related fractures are usually detected early. So far there is no defined age up to which fractures have to be considered birth related. If unspecific bridging symptoms are present, they can be evaluated as an indication of birth trauma.

## Data Availability

Data sharing is not applicable to this article as no new data were created in this study.
